# Weekly primaquine for radical cure of patients with *Plasmodium vivax* malaria and glucose-6-phosphate dehydrogenase deficiency

**DOI:** 10.1371/journal.pntd.0011522

**Published:** 2023-09-06

**Authors:** Walter R. J. Taylor, Niamh Meagher, Benedikt Ley, Kamala Thriemer, Germana Bancone, Ari Satyagraha, Ashenafi Assefa, Krisin Chand, Nguyen Hoang Chau, Mehul Dhorda, Tamiru S. Degaga, Lenny L. Ekawati, Asrat Hailu, Mohammad Anwar Hasanzai, Mohammad Nader Naddim, Ayodhia Pitaloka Pasaribu, Awab Ghulam Rahim, Inge Sutanto, Ngo Viet Thanh, Nguyen Thi Tuyet-Trinh, Naomi Waithira, Adugna Woyessa, Arjen Dondorp, Lorenz von Seidlein, Julie A. Simpson, Nicholas J. White, J. Kevin Baird, Nicholas P. Day, Ric N. Price

**Affiliations:** 1 Mahidol Oxford Tropical Medicine Research Unit, Faculty of Tropical Medicine, Mahidol University, Bangkok, Thailand; 2 Centre for Tropical Medicine and Global Health, Nuffield Department of Medicine, University of Oxford, Oxford, United Kingdom; 3 Centre for Epidemiology and Biostatistics, Melbourne School of Population and Global Health, University of Melbourne, Melbourne, Australia; 4 Department of Infectious Diseases University of Melbourne, at the Peter Doherty Institute for Infection and Immunity, Melbourne, Australia; 5 Global and Tropical Health Division, Menzies School of Health Research and Charles Darwin University, Darwin, Australia; 6 Shoklo Malaria Research Unit, Mahidol-Oxford Tropical Medicine Research Unit, Faculty of Tropical Medicine, Mahidol University, Mae Sot, Thailand; 7 Eijkman Institute of Molecular Biology, Jakarta, Indonesia.8. Ethiopian Public Health Institute, Addis Ababa, Ethiopia; 8 Ethiopian Public Health Institute, Addis Ababa, Ethiopia; 9 Oxford University Clinical Research Unit, Faculty of Medicine, Universitas Indonesia, Jakarta, Indonesia; 10 Oxford University Research Unit, Hospital for Tropical Diseases, Ho Chi Minh City, Vietnam; 11 College of Medicine & Health Sciences, Arbaminch University, Arbaminch, Ethiopia; 12 College of Health Sciences, Addis Ababa University, Addis Ababa, Ethiopia; 13 Health Protection and Research Organisation, Kabul, Afghanistan; 14 Universitas Sumatera Utara, Medan, Indonesia; 15 Nangarhar Medical Faculty, Nangarhar University, Ministry of Higher Education, Jalalabad, Afghanistan; 16 Health and Social Development Organization, Kabul, Afghanistan; 17 Faculty of Medicine, Universitas Indonesia, Jakarta, Indonesia; University of Florida, UNITED STATES

## Abstract

**Background:**

The World Health Organization recommends that primaquine should be given once weekly for 8-weeks to patients with *Plasmodium vivax* malaria and glucose-6-phosphate dehydrogenase (G6PD) deficiency, but data on its antirelapse efficacy and safety are limited.

**Methods:**

Within the context of a multicentre, randomised clinical trial of two primaquine regimens in *P*. *vivax* malaria, patients with G6PD deficiency were excluded and enrolled into a separate 12-month observational study. They were treated with a weekly dose of 0.75 mg/kg primaquine for 8 weeks (PQ8W) plus dihydroartemisinin piperaquine (Indonesia) or chloroquine (Afghanistan, Ethiopia, Vietnam). G6PD status was diagnosed using the fluorescent spot test and confirmed by genotyping for locally prevalent G6PD variants. The risk of *P*. *vivax* recurrence following PQ8W and the consequent haematological recovery were characterized in all patients and in patients with genotypically confirmed G6PD variants, and compared with the patients enrolled in the main randomised control trial.

**Results:**

Between July 2014 and November 2017, 42 male and 8 female patients were enrolled in Afghanistan (6), Ethiopia (5), Indonesia (19), and Vietnam (20). G6PD deficiency was confirmed by genotyping in 31 patients: Viangchan (14), Mediterranean (4), 357A-G (3), Canton (2), Kaiping (2), and one each for A^-^, Chatham, Gaohe, Ludhiana, Orissa, and Vanua Lava. Two patients had recurrent *P*. *vivax* parasitaemia (days 68 and 207). The overall 12-month cumulative risk of recurrent *P*. *vivax* malaria was 5.1% (95% CI: 1.3–18.9) and the incidence rate of recurrence was 46.8 per 1000 person-years (95% CI: 11.7–187.1). The risk of *P*. *vivax* recurrence was lower in G6PD deficient patients treated with PQ8W compared to G6PD normal patients in all treatment arms of the randomised controlled trial. Two of the 26 confirmed hemizygous males had a significant fall in haemoglobin (>5g/dl) after the first dose but were able to complete their 8 week regimen.

**Conclusions:**

PQ8W was highly effective in preventing *P*. *vivax* recurrences. Whilst PQ8W was well tolerated in most patients across a range of different G6PD variants, significant falls in haemoglobin may occur after the first dose and require clinical monitoring.

**Trial registration:**

This trial is registered at ClinicalTrials.gov (NCT01814683).

## Author summary

Fifty patients with *P*. *vivax* malaria and glucose-6-phosphate dehydrogenase (G6PD) deficiency were given the WHO-recommended, primaquine regimen of 0.75 mg/kg body weight once per week for 8 weeks. Treatment was highly effective and well tolerated in most patients. Two patients had a significant (>5g/dl) fall in haemoglobin and one was hospitalised for intravenous fluids; both were able to complete the primaquine regimen.

## Introduction

*Plasmodium vivax* forms dormant liver stages (hypnozoites) that can reactivate periodically, causing recurrent blood stage infections termed relapses. Overall, relapses are estimated to cause up to 80% of recurrent patent *P*. *vivax* infections across a range of endemic settings [1–3], and can be associated with significant morbidity and mortality [4–6]. In endemic areas a high proportion of individuals infected with *P*. *vivax* also have asymptomatic *P*. *vivax* parasitaemia that sustains malaria transmission despite conventional approaches to malaria control [7].

The only drugs currently available for treating hypnozoites are the 8-aminoquinoline drugs primaquine and tafenoquine, both of which cause dose-dependent acute hemolytic anemia in individuals with glucose-6-phosphate dehydrogenase deficiency (G6PDd) [8,9]. X-linked G6PDd is very common in areas where malaria is or was endemic. G6PD is the first enzyme in the pentose phosphate shunt pathway which produces NADPH required for regenerating reduced glutathione (GSH), and a healthy reducing red blood cell cytosol. Acute hemolysis in G6PD deficient individuals may also be triggered by fava beans, chemical oxidants, or infection [10,11].

In the 1950s, clinical trials demonstrated that the hemolytic risk of primaquine in G6PDd patients could be mitigated by administering primaquine as a weekly regimen for 8 weeks rather than daily for 14 days [12]. The World Health Organization (WHO) subsequently endorsed the 8 week regimen (PQ8W) for patients with G6PDd in all endemic regions [13]. Despite this long-standing recommendation, only four clinical trials have assessed the tolerability, safety and efficacy of PQ8W, three of which assessed G6PD activity reporting a total of 59 G6PD deficient patients exposed to weekly primaquine [14–17]; S1 Table

As part of a large multicentre, randomised controlled clinical trial of primaquine in G6PD normal patients with vivax malaria (IMPROV) [18], patients diagnosed with G6PDd were excluded from that main trial and enrolled into an observational study to assess the safety and efficacy of PQ8W over 12 months of follow up.

## Materials and methods

### Ethics statement

Ethics approval was obtained from the following national and local committees and authorities: The Human Research Ethics Committee of the Northern Territory Department of Health (HREC), Australia, the Islamic Republic of Afghanistan, Ministry of Public Health, Institutional Review Board, Afghanistan, the National Research Ethics Review Committee (NRERC), Ethiopia, the Scientific & Ethical Review Committee (SERC), Ethiopian Public Health Institute, Ethiopia, the Food Medicine and Health Care Administration and Control Authority (FMHACA), Ethiopia, the Health Research Ethics Committee of the Faculty of Medicine University of Indonesia, Indonesia, the Indonesian Food and Drug Agency (BPOM), Indonesia, the Oxford Tropical Research Ethics Committee (OxTREC), UK, and the Ministry of Health Evaluation Committee on Ethics in Biomedical Research, Vietnam.

### Trial design, study site, conduct and ethics

The clinical trial design and methodology have been described previously [18,19]. Briefly, patients presenting with confirmed acute uncomplicated vivax malaria who presented with fever or fever history within the previous 48 hours, were aged ≥ 6 months, weighed ≥ 5 kg and had a haemoglobin (Hb) concentration ≥ 9 g/dL, were enrolled at sites in Afghanistan, Ethiopia, Indonesia and Vietnam (S2 Table). Patients diagnosed with G6PDd, by the fluorescent spot test (FST), who gave written consent or assent (12–17 years) with legal guardian consent were enrolled into an observational substudy. Patients were excluded from the observational study if they were pregnant or lactating, unable to tolerate oral treatment, had signs of severe malaria, reported previous hemolytic episodes, had a blood transfusion within the previous 90 days, known hypersensitivity to study drugs, or were taking drugs known to cause hemolysis in G6PDd individuals or interfere with the pharmacokinetics of the study drugs.

G6PDd patients were treated with supervised primaquine (blister-packed 7.5 & 15 mg tablets, Centurion Laboratories, Vadodara, India), at a target dose of 0.75mg base/kg per week (S4 Table), started on either Day 0 or Day 1. In Afghanistan, Ethiopia and Vietnam patients were also treated with chloroquine (CQ, target total dose of 25 mg base/kg over 48 hours) whilst Indonesian patients received standard dosing with dihydroartemisinin-piperaquine by weight, 3 doses over 48h following national guidelines (S5 Table). All patients were observed for one hour and, if they vomited, the study clinicians had the option of redosing with both drugs, or omitting the primaquine.

At enrolment, a medical history was taken, a physical examination was performed, and antimalarial treatment initiated. Patients were reviewed weekly for the first 8 weeks and then monthly for one year. At each visit, blood was taken and Giemsa stained for microscopic examination for peripheral parasitaemia, and Hb concentration (HemoCue AB, Ängelholm, Sweden). Blood films were declared negative if no parasites were detected after examining 200 high power fields (1,000× magnification) on the thick film.

The primary efficacy outcome was the incidence rate of all recurrent vivax parasitaemia over 12 months of follow up, i.e. the total number of detected *P*. *vivax* recurrences divided by the total person time of follow up, expressed as the number of recurrences per 1000 person-years (p-y). The secondary efficacy outcomes included the incidence risk of *P*. *vivax* parasitaemia over 12 months, the incidence rate and incidence risk of any parasitaemia (i.e., any *Plasmodium* species), and the incidence risk of *P*. *vivax* parasitaemia by D28. The efficacy of blood stage treatment examined the proportions of patients with *P*. *vivax* parasitaemia, and fever on D0-2.

Haematological safety outcome measures included: the incidence risk of severe anemia (Hb <7g/dL) and/or blood transfusion, and an acute drop in Hb >5g/dL over the first 56 days of follow up, the median fall in Hb concentration from baseline to D3 and D7, the nadir Hb concentration (defined as minimum Hb concentration recorded over 56 days), the median time to nadir Hb, the maximal fractional change of Hb concentration (calculated from the difference in Hb at nadir from baseline), and the proportion of patients who achieved complete Hb recovery (D90 Hb > baseline Hb concentration).

Adverse events (AE) were graded using the National Institute of Allergy and Infectious Diseases Table for grading the severity of Adult and Pediatric adverse events. Solicited gastrointestinal symptoms in routine questionnaires included nausea, vomiting and abdominal pain between D0 and D13. Any AE leading to study drug withdrawal, and serious AEs over 12 months were also recorded, irrespective of their relationship to study drugs.

Ethics approvals were obtained from the relevant national and local committees and authorities, the Oxford University Tropical Ethics Committee and the Human Research Ethics Committee of the Northern Territory Department of Health, Australia (S3 Table).

### G6PD deficiency assessment and genotyping

At the two Indonesian sites, patients with an FST result indicating G6PD deficiency had their enzyme activities quantified by spectrophotometry (Trinity Biotech, Ireland), processed within 7 days of sampling [20], and absolute spectrophotometry readings (U/gHb) converted into percentage activity of the adjusted male median (AMM), derived separately for each site [21].

Patient’s G6PDd status was confirmed by genotyping for local variants known to be associated with reduced enzyme activity. DNA from these Indonesian samples was extracted using QIAamp DNA mini kit (Qiagen, Germany) and genotyped first by PCR/RFLP for regional variants including Viangchan, Chatham, Mahidol, Vanua Lava, Orissa, Kaiping, Union, and Coimbra. If these variants were not detected, samples were sequenced from exons 3−13, as described previously [22]. For the patients enrolled in Afghanistan, Vietnam and Ethiopia, DNA was extracted from whole blood using column kits (Favorgen Biotech, Taiwan) and G6PD genotypes determined by full gene sequencing of exons 2−13 [23]. The electrophoregrams were visualized and analysed with the Qiagen CLC Genomics Workbench (Qiagen, Germany). Nucleotide sequences were compared to G6PD sequences in GenBank (accession No. X55448) to identify mutations.

### Statistical methods & data management

Data were entered from standardised case record forms and analysed using Stata v16.0 (StataCorp, College Station, TX). Negative binomial regression adjusted for follow up time was used to estimate the incidence rate of recurrence outcomes and Kaplan Meier survival estimates were used to assess the incidence risk of recurrent parasitaemia. Descriptive statistics are presented for haematological outcomes. All patients who received at least one dose of study drug were included in the efficacy and safety analyses. Estimates of safety and efficacy outcomes are provided with 95% confidence intervals but no statistical comparison is made between these estimates in the patients treated with weekly primaquine and those in main randomised trial. Since only eligible patients agreeing to be enrolled into the IMPROV study were tested for G6PD deficiency, reliable estimates of the background prevalence of G6PD at each of the sites were not available. Handling of missing data and adjudication of outcomes are described in detail in the *a priori* statistical analysis plan (S1 Text). To account for potential bias by inclusion of G6PD normal patients enrolled in error, a subgroup analysis was conducted in patients in whom G6PDd was confirmed by genotyping.

## Results

**Fig 1:**
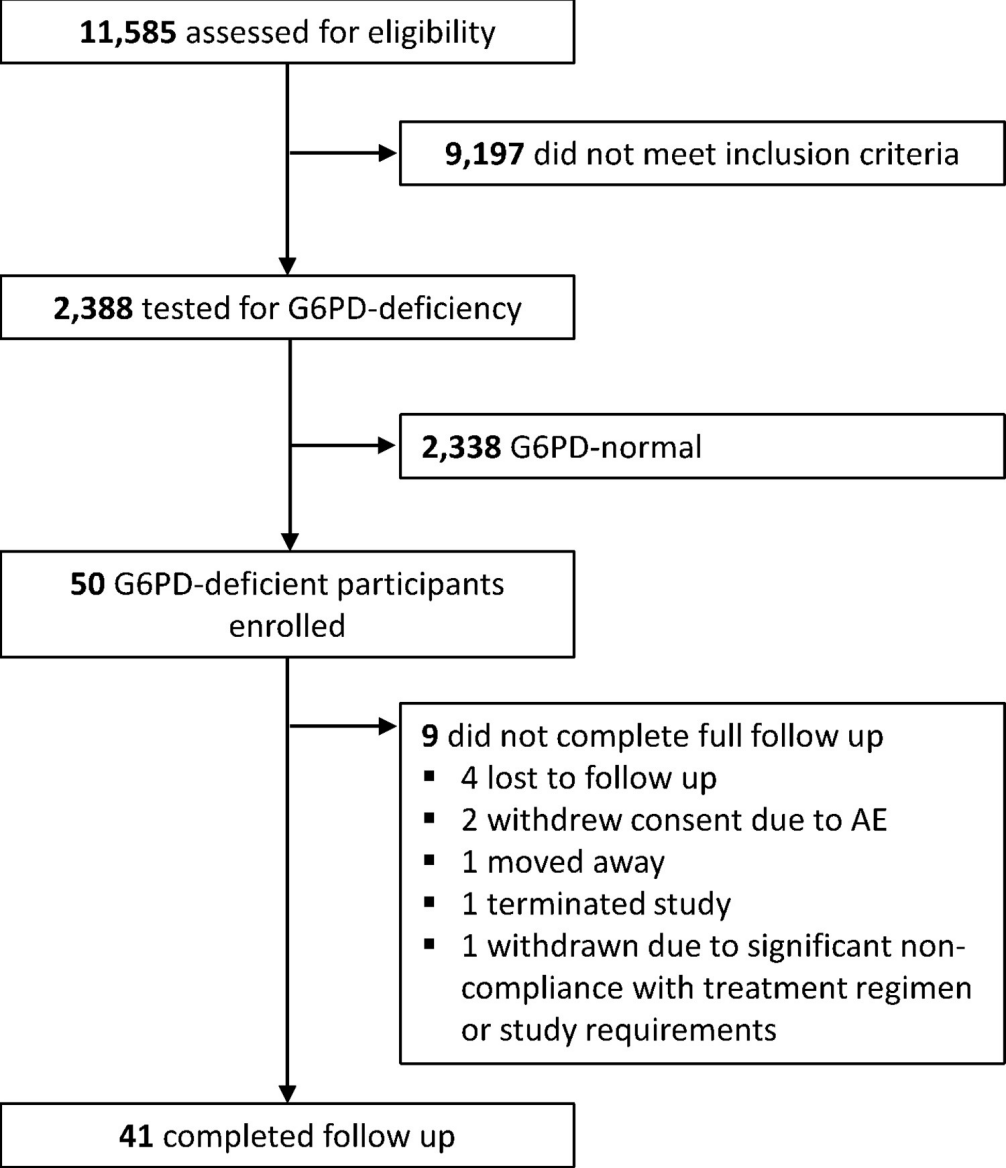
Trial profile.

Between July 20, 2014, and November 25, 2017, 50 patients (42 [84%] males) with G6PDd and microscopy confirmed *P*. *vivax* malaria were enrolled, 49 with *P*. *vivax* mono infections and 1 with mixed infection of *P*. *vivax* and *P*. *falciparum* (Fig 1 and Table 1). The median age of patients was 23 (interquartile range [IQR]: 14−38) years, of whom 36 (72%) were adults, 10 (20%) were aged 5−15 and 4 (8%) were <5 years old. The geometric mean baseline parasitaemia was 2,947 parasites μL^-1^ (95% normal range: 70–25,000) and was highest in Ethiopia (6,156 μL^-1^) and lowest in Afghanistan (1,119 μL^-1^); (S6 Table). Gametocytaemia was detected in 37 (74%) patients. The median baseline Hb concentration was 13.4 g/dL (Range: 9.2–17.8) with 3 patients (1 female and 2 males) having an Hb <10 g/dL. Compared to G6PD normal patients enrolled into the randomised treatment arms, G6PDd patients were more likely to be enrolled in Vietnam, be male, and to have a lower parasite density. Overall, 92% (46/50) patients completed all 8-doses of primaquine; the median total mg/kg dose administered was 6.0 mg/kg (range 4.3 − 7.8). In total, 40 (80%) patients completed 12 months of follow up.

**Table 1:** Baseline characteristics of patients enrolled into the study.

	All patients diagnosed with G6PD deficiency by FST	Subgroup of patients with G6PD deficiency confirmed by genotyping	G6PD normal patients enrolled in the randomised trial		
	PQ 8 weeks	PQ 8 weeks	Placebo	PQ7days	PQ14days
	N = 50	N = 31	N = 464	N = 935	N = 937
Patients enrolled N (%)					
Afghanistan	6 (12.0%)	5 (16.1%)	83 (17.9%)	173 (18.5%)	175 (18.6%)
Ethiopia	5 (10.0%)	4 (12.9%)	114 (24.5%)	234 (25.0%)	232 (24.8%)
Indonesia	19 (38.0%)	6 (19.4%)	203 (43.7%)	400 (42.8%)	397 (42.3%)
Vietnam	20 (40.0%)	16 (51.6%)	64 (13.8%)	128 (13.7%)	133 (14.2%)
Age in years, median (IQR) [range]	23.0 (14.0–38.0) [0.0–60.0]	20.0 (14.0–36.0) [0.8–60.0]	17.0 [10.0–28.0]	16.0 [10.0–25.0]	16.0 [10.0–26.0]
Age category months (m) or years(y)					
6–11 m	1 (2.0%)	1 (3.2%)	1 (0.2%)	1 (0.1%)	1 (0.1%)
≥1 & <5 y	3 (6.0%)	2 (6.5%)	28 (6.0%)	60 (6.4%)	63 (6.7%)
≥5 & <15 y	10 (20.0%)	7 (22.6%)	163 (35.1%)	358 (38.3%)	335 (35.8%)
≥15 y	36 (72.0%)	21 (67.7%)	272 (58.6%)	516 (55.2%)	538 (57.4%)
Sex					
Male	42 (84.0%)	26 (83.9%)	296 (63.8%)	563 (60.2%)	608 (64.9%)
Female	8 (16.0%)	5 (16.1%)	168 (36.2%)	372 (39.8%)	329 (35.1%)
Weight in kg median (IQR)	51.0 (36.0–60.3)	48.8 (35.5–60.0)	47.0 [26.4–57.5]	46.2 [26.0–57.0]	46.8 [25.3–56.6]
Weight category					
10–22 kg	7 (14.0%)	5 (16.1%)	3 (0.6%)	8 (0.9%)	6 (0.6%)
23–34 kg	4 (8.0%)	2 (6.5%)	89 (19.2%)	180 (19.3%)	180 (19.2%)
34–45 kg	6 (12.0%)	5 (16.1%)	67 (14.4%)	136 (14.5%)	143 (15.3%)
46+ kg	33 (66.0%)	19 (61.3%)	64 (13.8%)	135 (14.4%)	121 (12.9%)
*P*. *vivax* parasites/uL geometric mean (95% normal range)	2947 (70–25000)	2555 (67–67500)	3702 (150–53057)	3330 (144–55000)	3426 (137–50000)
Gametocytaemia	37 (74.0%)	23 (74.2%)	335 (72.2%)	713 (76.3%)	704 (75.1%)
Gametocytes/uL geometric mean (95% normal range)	138.2 (15.0–3593)	98 (15–1204)	211 (15–3037)	200 (15–3815)	198 (15–2545)
Temperature(°C) Mean [SD]	37.7 [1.2]	37.8 (1.3)	37.9 [1.2]	37.8 [1.2]	37.8 [1.2]
Fever (Axillary >37.5C or Oral >38C) N (%)	25 (50.0%)	17 (54.8%)	267 (57.9%)	537 (57.9%)	538 (57.5%)
Hemoglobin (g/dL) Mean [SD]†	13.5 [1.9]	13.1 [1.8]	13.0 [1.7]	13.0 [1.8]	12.9 [1.7]
Hb <10 g/dL	3 (6.0%)	2 (6.5%)	19 (4.1%)	30 (3.2%)	40 (4.3%)

The baseline characteristics of patients enrolled at each site are presented in S6 Table

### G6PD activity and genotyping

Genotyping of G6PD variants was conducted on all but one patient from Afghanistan who had a missing blood sample. Seventeen patients were wild type at the loci tested (See S1 Data). One Ethiopian male had the AF2 (202G>A) genotype, consistent with the A+ variant, and was categorized as unconfirmed G6PDd. A known G6PDd variant was confirmed in the remaining 31 (62%) patients.

In South Sumatra, the G6PD activity of the six patients diagnosed with G6PD deficiency by FST ranged from 1.4 to 52% (S1 Data); their genotypes were Viangchan (3 hemizygotes), Orissa (1 hemizygote), Chatham (1 heterozygote) and Vanua Lava (1 heterozygote). In North Sumatra, 8 of the 13 G6PD deficient patients diagnosed by FST had enzyme activities >30% activity (68−119%) whilst the activity of the remaining five patients could not be determined because their samples arrived in the reference laboratory >20 days after collection. All 13 patients were screened for the 4 common local variants (Chatham, Viangchan, Mahidol and Vanua Lava), but none were detected.

Of the 31 patients enrolled in Ethiopia, Afghanistan and Vietnam, a G6PD variant was identified in 22 hemizygous males and 3 heterozygous females (Table 2).

**Table 2:** G6PD variants identified in patients enrolled into the study.

	Afghanistan	Ethiopia*	Indonesia	Vietnam	Total
	N = 6	N = 5	N = 19	N = 20	N = 50
357A-G		3 (60.0%)			3 (6.0%)
Asahri (AF1)		1 (20.0%)			1 (2.0%)
Canton				2 (10.0%)	2 (4.0%)
Chatham			1 (5.3%)		1 (2.0%)
Gaohe				1 (5.0%)	1 (2.0%)
Kaiping				2 (10.0%)	2 (4.0%)
Ludhiana	1 (16.7%)				1 (2.0%)
Mediterranean	4 (66.7%)				4 (8.0%)
Orissa			1 (5.3%)		1 (2.0%)
Viangchan			3 (15.8%)	11 (55.0%)	14 (28.0%)
Vanua Lava			1 (5.3%)		1 (2.0%)
Confirmed G6PDd	5 (83.3%)	4 (80.0%)	6 (31.6%)	16 (80%)	31 (62.0%)
Hemizygous males	5	4	4	13	26
Heterozygous females	0	0	2	3	5
Unconfirmed	1 (16.7%)	1 (20.0%)*	13 (68.4%)	4 (20.0%)	19 (38.0%)

*1 male in Ethiopia genotyped as AF2 (202G>A) and was categorized as A+ and not confirmed as deficient. Enzyme activities are presented in S1 Data.

### Efficacy

The initial response to treatment was rapid in all patients. After 24 hours, 88% (44/50) were aparasitaemic and 92% (45/49) were afebrile. By 48 hours, all patients had cleared their peripheral parasitaemia and were afebrile. Two Vietnamese patients had recurrent symptomatic *P*. *vivax* parasitaemia on D68 and D207. Both were males with confirmed hemizygous G6PDd; they had completed their PQ8W and received total primaquine doses of 5.5 and 5.9 mg/kg, respectively.

The incidence rate of *P*. *vivax* parasitaemia was 46.8 recurrences/1000 p-y (95% CI: 11.7 − 187.1), and the 12-month incidence risk of recurrence was 5.1% (95% CI: 1.3–18.9%). One patient from Vietnam also had asymptomatic *P*. *falciparum* documented for 3 consecutive weeks. The corresponding incidence risks and rate of *P*. *vivax* parasitaemia were higher in G6PD normal patients enrolled into all arms of the randomised controlled trial (Table 3).

**Table 3:** Efficacy endpoints.

		All patients diagnosed with G6PD deficiency by FST	Subgroup of patients with G6PD deficiency confirmed by genotyping	G6PD normal patients enrolled in the randomised trial		
		PQ 8 weeks	PQ 8 weeks	Placebo	PQ7days	PQ14days
		N = 50	N = 31	N = 464	N = 935	N = 937
Incidence Rates per 1000 person years (95%CI)						
	Total patient days of follow-up (days) [1]	15,613	10,235	138,712	278,655	285,652
1 Year	Symptomatic *P*. *vivax* parasitaemia	46.8 (11.7 − 187.1)	71.4 (17.9–285.4)	960 (830–1080)	180 (150–210)	160 (130–180)
	Any *P*. *vivax* parasitaemia	46.8 (11.7 − 187.1)	71.4 (17.9–285.4)	1320 (1150–1480)	230 (190–270)	190 (150–230)
	Any *P*. *falciparum* parasitaemia	70.2 (3.9–1263.3)	107.1 (6.1–1885.3)	100 (50–150)	150 (110–190)	100 (70–140)
	Parasitaemia due to any species	117.0 (29.9–457.0)	178.4 (46.8–680.6)	1420 (1250–1590)	380 (320–440)	300 (240–350)
Incidence Risks (95%CI)						
Day 28	Any *P*. *vivax* parasitaemia	0%	0%	1.7% (0.8–3.6)	0.23% (0.06–0.93)	0.33% (0.10–1.00)
Day 42	Any *P*. *vivax* parasitaemia	0%	0%	7.9% (5.6–11.0)	0.87% (0.41–1.81)	0.82% (0.39–1.72)
1 Year	Symptomatic *P*. *vivax* parasitaemia	5.1% (1.3–18.9)	7.9% (2.0–28.1)	48.7% (43.4–54.4)	14.3% (11.8–17.3)	12.7% (10.2–15.8)
	Any *P*. *vivax* parasitaemia	5.1% (1.3–18.9)	7.9% (2.0–28.1)	58.40% (53.3–63.6)	16.0% (13.4–19.1)	14.8% (12.1–17.9)
	Any *P*. *falciparum* parasitaemia	2.1% (0.3–13.9)	3.2% (0.5–20.8)	11.4% (7.7–16.8)	8.67% (6.80–11.03)	7.22% (5.51–9.45)
	Parasitaemia due to any species	7.2% (2.4 − 20.9)	11.2% (3.7–31.0)	1.4% (1.3–1.6)	0.38% (0.32–0.44)	0.30% (0.24–0.35)

### Haematological profile

**Fig 2:**
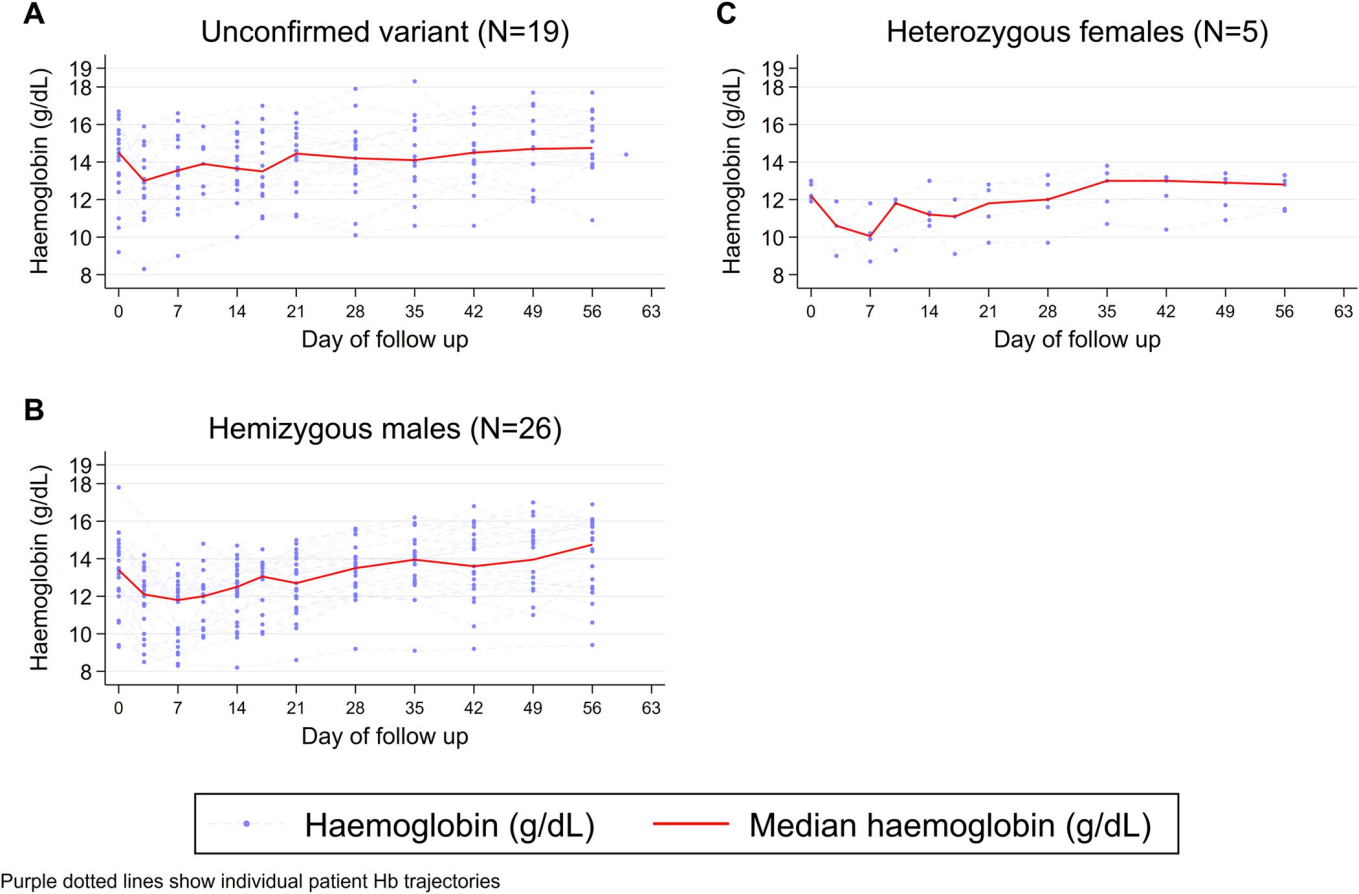
Changes in haemoglobin concentrations (g/dL) over time in all patients identified as G6PD deficient by the fluorescent spot test. Footnote: Purple dotted lines show individual patient Hb profiles.

Overall, the median Hb concentration fell after starting treatment with the lowest concentration reported on D7 (12.2 g/dL, range 8.3–16.6). The Hb concentration rose thereafter to exceed the median baseline Hb on D28 (Fig 2). The median fall in Hb was -1.5 g/dL (range -4.3 to 2.5) on D3 and -1.6 g/dL (-5.1 to 3.2) on D7. The median maximal change in Hb was -1.8 g/dl (range -5.6 to 0), representing a median maximal fractional fall in Hb of -13.0% (range -33.3 to 0%).

**Fig 3:**
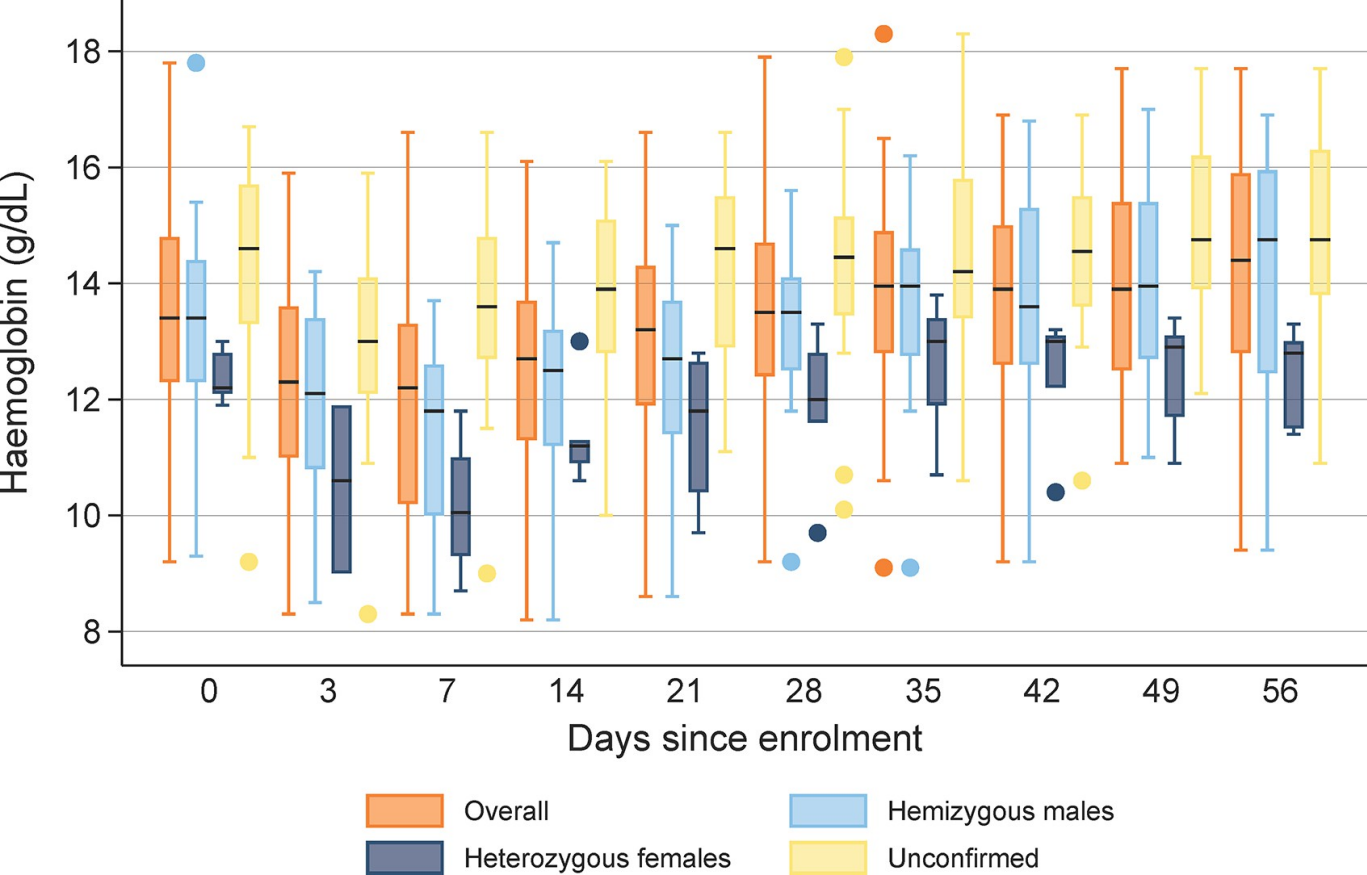
Distribution of haemoglobin concentrations (g/dL) over time during follow up by G6PD status. Footnote: Boxes represent 25^th^ and 75^th^ percentiles.

The Hb concentrations over time by G6PD status are shown in Fig 3. The median maximal fall in Hb was -2.0 g/dL (range -5.6 to 0) in the 25 confirmed hemizygous males, -2.0 g/dL (range -4.1 to -1.1) in the 5 confirmed heterozygous females, and -1.7 g/dL (range -3.7 to 0) in the 16 patients without confirmed G6PD deficiency. The corresponding median maximal fall in Hb by D28 in G6PD normal patients enrolled into the randomised controlled trial were: -0.9 g/dL (range -4.9 to 0) in the placebo arm, -0.8 g/dL (range -5.6 to 0) in patients treated with 7 day primaquine regimen (1mg/kg/day) and -1.0 g/dL (range -5.5 to 0) in patients treated with the 14 day primaquine regimen (0.5 mg/kg/day); Table 4.

**Table 4:** Hemoglobin Profile by Treatment Arm.

	All patients diagnosed with G6PD deficiency by FST	Subgroup of patients with G6PD deficiency confirmed by genotyping	G6PD normal patients enrolled in the randomised trial		
	PQ 8 weeks	PQ 8 weeks	Placebo	PQ7days	PQ14days
Number of patients	N = 50	N = 31	N = 464	N = 935	N = 937
Incidence risk of severe anemia (<7 g/dL) or transfusion within 365 days, (95% CI) [N]	[N = 0]	[N = 0]	[N = 0]	0.40 (0.13, 1.30) [N = 3]	0.11 (0.02, 0.77) [N = 1]
Hb drop >5g/dL within 7 days of initial treatment, N (%)	1 (2.0)	1 (3.2)	0 (0.0)	0 (0.0)	1 (0.1)
Hb nadir within 28 (G6PDn) or 56 (G6PDd) days of treatment initiation, g/dL, median [range]	11.85 [8.2–15.1]	11.55 [8.2–13.2]	11.9 [7.8–16.6]	11.9 [6.9–17.9]	11.8 [8.1–17.5]
Time to Hb nadir, days, median (interquartile range)	7 (3–7)	7 (3–7)	3 (3–7)	3 (3–13)	3 (3–13)
Maximal fall in Hb, g/dL, median [range]	-1.8 [-5.6–0]	-2.0 [-5.6–0]	-0.9 [-4.9–0]	-0.8 [-5.6–0]	-1.0 [-5.6–0]
Maximal fractional fall in Hb, %, median [range]	-13.0 [-33.3–0]	-16.0 [-33.3–0]	-7.1 [-36.1–0]	-6.4 [-40.5–0]	-7.4 [-33.6–0]
Day 0					
Number of Patients	50	31	464	934	937
Hb on day 0, g/dL, median [range]	13.4 [9.2–17.8]	13.2 [9.3–17.8]	13.1 [9.0–18.5]	12.8 [9.0–19.0]	13.0 [9.0–18.3]
Day 7					
Number of patients	47	29	447	882	888
Hb on day 7, g/dL, median [range]	12.2 [8.3–16.6]	11.8 [8.3–13.7]	12.7 [8.5–17.3]	12.85 [7.8–20.0]	12.8 [7.7–17.9]
Change in Hb between D0 and D7, g/dL, median [range]	-1.6 [-5.1–3.2]	-1.9 [-5.1–0.3]	-0.3 [-4.9–5.2]	-0.1 [-4.1–4.8]	-0.1 [-4.3–4.9]
Absolute drop between D0 and D7 >5 g/dL, N (%)	1 (2.1)	1 (3.4)	0 (0.00)	0 (0.00)	0 (0.00)
Fractional % change in Hb between D0 and D7 (median [range])	-11.9 [-33.3–23.9]	-16.0 [-33.3–3.2]	-2.2 [-32.0–43.0]	0.7 [-29.5–38.4]	-0.8 [-33.6–47.1]
Fractional drop between D0 and D7 >25%, N (%)	4 (8.5)	4 (13.8)	2 (0.45)	9 (1.02)	2 (0.23)
Day 13 (G6PDn) and Day 14 (G6PDd)					
Number of patients	46	30	438	852	866
Hb on day 13 or 14, g/dL, median [range]	12.8 [8.2–16.1]	12.4 [8.2–14.7]	12.9 [8.9–18.3]	12.7 [9.3–18.2]	12.9 [9.0–17.6]
Change in Hb between D0 and D13/D14, g/dL, median [range]	-0.7 [-5.6–3.0]	-0.9 [-5.6–3.0]	0.0 [-4.0–4.0]	-0.1 [-5.1–5.1]	0.1 [-4.6–5.0]
Absolute drop between D0 and D13/D14 >5 g/dL, N (%)	1 (2.2)	1 (3.3)	0 (0.00)	1 (0.12)	0 (0.00)
Fractional change in Hb between D0 and D13/D14, %, median [range]	-5.1 [-31.5–32.3]	-7.4 [-31.5–32.3]	0.0 [-23.2–39.6]	-0.7 [-30.0–49.0]	0.7 [-28.2–48.1]
Fractional drop between D0 and D13/D14 >25%, N (%)	2 (4.3)	2 (6.7)	0 (0.00)	3 (0.35)	2 (0.23)
Day 28					
Number of patients	47	30	396	817	813
Hb on day 28, g/dL, median [Range]	13.5 [9.2–17.9]	13.2 [9.2–15.6]	13.3 [8.9–17.9]	13.3 [9.6–18.0]	13.2 [8.7–17.9]
Day 42					
Number of patients	47	30	378	785	773
Hb on day 42, g/dL, median [Range]	13.9 [9.2–16.9]	13.3 [9.2–16.8]	13.5 [9.1–17.8]	13.6 [9.8–18.9]	13.6 [9.3–19.5]

**Fig 4:**
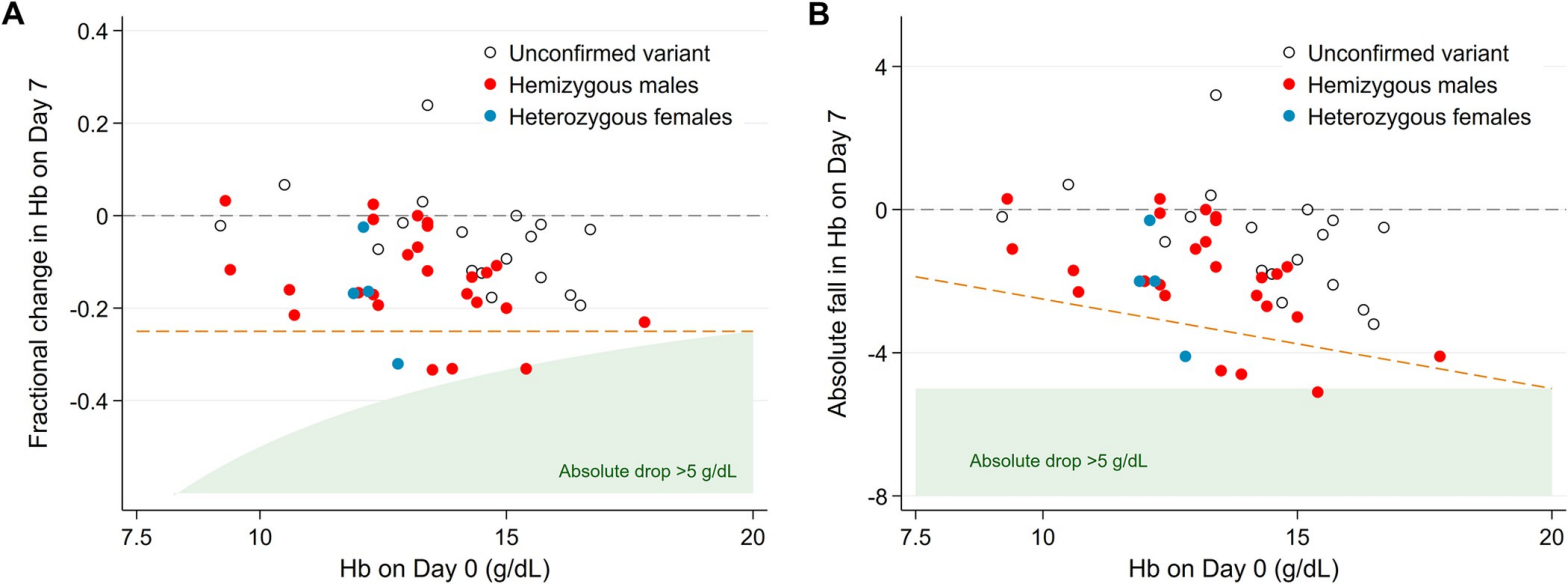
Relationship between haemoglobin at baseline and day 7 as A) fractional change and B) absolute change, by G6PD status.

Two patients had falls in Hb exceeding 5 g/dL (Fig 4). A 19-year old male with the Mediterranean (C563T) variant had a fall in Hb from 17.8 g/dL at baseline to 12.2 g/dL on D14 (fractional fall of 31%) but was otherwise well. The other patient was a 28-year male with Kaiping variant who had a fall in Hb from 15.4 g/dL to 10.3 g/dl on D7 (33% fall). He defaulted on his 3rd and 4th doses, but by D28 his Hb had risen to 13.8 g/dL and he resumed treatment without further complications. Four patients had Hb falls between 4.1 and 4.6 g/dL (1 Viangchan hemizygous male, 1 Kaiping hemizygous male, 1 unconfirmed G6PDd male, and 1 Vanua Lava heterozygous female). The lowest recorded Hb concentration was 8.2 g/dL. No patients required a blood transfusion.

### Tolerability

No patient vomited their medication within 1 hour of administration. During the first week (Table 5), abdominal pain was reported by 20.4% (10/49) of patients, nausea by 22.4% (11/49), and vomiting by 2.0% (1/50). The proportion of G6PD deficient patients reporting symptoms following PQ8W was similar to that in the G6PD normal patients treated in the placebo arm of the main trial (Table 5). There was one SAE reported in a 16-year old male with the Kaiping variant, whose Hb fell from 13.5 g/dL at baseline to 9.7 g/dL (28.1%) on D3, and 9.0 g/dL (33.3%) on D7 associated with general malaise, anorexia and reduced oral intake requiring hospital admission for intravenous fluids. On day 3, the colour of his urine was dark (Hillman score of 5). By day 14, his Hb was 12.0 g/dL and he recommenced weekly primaquine without further incident.

**Table 5:** Symptoms elicited from symptom questionnaires.

	All patients diagnosed with G6PD deficiency by FST	Subgroup of patients with G6PD deficiency confirmed by genotyping	G6PD normal patients enrolled in the randomised trial		
Symptom	PQ 8 weeks	PQ 8 weeks	Placebo	PQ7days	PQ14days
Any time during follow up	N = 50	N = 31	N = 464	N = 935	N = 937
Vomiting Study Drug within 1 hour, N (%)	0 (0.0)	0 (0.0)	11 (2.4)	21 (2.2)	20 (2.1)
Week 1 (D1, 2, 3, 7)	N = 49	N = 31	N = 461	N = 926	N = 933
Vomiting, n (%)	1 (2.0)	1 (3.2)	55 (11.9)	149 (16.1)	114 (12.2)
Headache, n (%)	23 (46.9)	10 (32.3)	207 (44.9)	430 (46.4)	426 (45.7)
Nausea, n (%)	11 (22.4)	5 (16.1)	145 (31.5)	315 (34.0)	296 (31.7)
Diarrhoea, n (%)	2 (4.1)	1 (3.2)	16 (3.5)	49 (5.3)	26 (2.8)
Rash, n (%)	1 (2.0)	0 (0.0)	7 (1.5)	13 (1.4)	16 (1.7)
Poor appetite, n (%)	17 (34.7)	8 (25.8)	167 (36.2)	365 (39.4)	352 (37.7)
Abdominal pain, n (%)	10 (20.4)	3 (9.7)	121 (26.3)	322 (34.8)	255 (27.3)
Myalgia / Arthralgia, n (%)	11 (22.4)	3 (9.7)	112 (24.3)	224 (24.2)	211 (22.6)
Fever, n (%)	12 (24.5)	10 (32.3)	123 (26.7)	251 (27.1)	276 (29.6)
Passing dark urine, n (%)	2 (4.1)	2 (6.5)	18 (3.9)	38 (4.1)	39 (4.2)
Dizziness, n (%)	5 (10.2)	3 (9.7)	66 (14.3)	144 (15.6)	136 (14.6)
Shortness of breath, n (%)	0 (0.0)	0 (0.0)	13 (3.0)	19 (2.1)	15 (1.6)
Itching, n (%)	0 (0.0)	0 (0.0)	9 (2.0)	16 (1.7)	19 (2.0)
Any gastrointestinal symptom*, n (%)	23 (46.9)	11 (35.5)	225 (48.8)	495 (53.5)	470 (50.4)
Week 2 (D10, 13, 14)	N = 49	N = 31	N = 452	N = 897	N = 911
Vomiting, n (%)	1 (2.0)	1 (3.2)	2 (0.4)	9 (1.0)	5 (0.6)
Headache, n (%)	4 (8.2)	2 (6.5)	17 (3.8)	51 (5.7)	46 (5.1)
Nausea, n (%)	2 (4.1)	1 (3.2)	8 (1.8)	25 (2.8)	23 (2.5)
diarrheal, n (%)	0 (0.0)	0 (0.0)	1 (0.2)	11 (1.2)	8 (0.9)
Rash, n (%)	1 (2.0)	0 (0.0)	2 (0.4)	7 (0.8)	2 (0.2)
Poor appetite, n (%)	4 (8.2)	2 (6.5)	13 (2.9)	37 (4.1)	20 (2.2)
Abdominal pain, n (%)	2 (4.1)	2 (6.5)	14 (3.1)	64 (7.1)	45 (4.9)
Myalgia / Arthralgia, n (%)	1 (2.0)	1 (3.2)	8 (1.8)	17 (1.9)	15 (1.7)
Fever, n (%)	3 (6.1)	2 (6.5)	8 (1.8)	15 (1.7)	25 (2.7)
Passing dark urine, n (%)	0 (0.0)	0 (0.0)	1 (0.2)	3 (0.3)	4 (0.4)
Dizziness, n (%)	0 (0.0)	0 (0.0)	5 (1.1)	16 (1.8)	13 (1.4)
Shortness of breath, n (%)	1 (2.0)	1 (3.2)	2 (0.4)	5 (0.6)	5 (0.6)
Itching, n (%)	0 (0.0)	0 (0.0)	3 (0.7)	3 (0.3)	1 (0.1)
Any gastrointestinal symptom*, n (%)	5 (10.2)	3 (9.7)	23 (5.1)	86 (9.6)	62 (6.8)

* Composite of any of the following: nausea, vomiting, anorexia, diarrheal or abdominal pain

## Discussion

Our study represents the largest study to date on the use of the 8-week primaquine regimen for the radical cure of *P*. *vivax* malaria in patients with G6PD deficiency. The regimen was highly effective. Only two patients (4%) had documented episodes of recurrent *P*. *vivax* parasitaemia, both occurring late in follow up.

Defining antirelapse efficacy of primaquine and tafenoquine is confounded by the frequency and timing of hypnozoite reactivation and recurrent parasitaemia, which vary with hypnozoite load, and parasite strains in a geographical region [24]. Estimates are further confounded by the inability to distinguish the origin of recurrent infections which can arise from recrudescences, reinfections or relapses [25], emphasizing the importance of conducting antirelapse trials with a suitable comparator arm [26]. In the current analysis, we employed a parallel placebo arm from the contemporaneous randomised trial involving G6PD-normal *P*. *vivax* patients from the same communities to estimate the safety and efficacy of PQ8W. In view of potential bias between patients in the G6PD deficient cohort and the main randomised treatment arms, formal statistical comparisons are not provided. The overall risk of recurrence after 12 months in G6PD deficient patients treated with PQ8W was 5.1%, 10 times lower than that reported in G6PD normal patients treated with blood schizonticidal antimalarials alone (49%) and 2–3 fold lower than that in G6PD normal patients treated with the same total dose of primaquine administered daily (13–16%); Table 3 [18]. Radical curative efficacy of 8-aminoquinolines is proportional to the total dose given [27]. The total dose of primaquine in the once weekly regimen (6mg/kg) is lower than that in the dose daily regimen (7mg/kg), and the last few doses are given after emergence of first relapses in tropical areas [24]. This interesting finding suggests that G6PD deficiency itself may contribute to radical curative efficacy, consistent with the protective effect against *P*. *vivax* malaria observed in patients with severe G6PDd [28,29] and the observation that the vivax parasitaemia in G6PD Mahidol is lower in G6PD patients compared to G6PD normal patients [30].

Few studies have estimated the efficacy of weekly primaquine. In the 1960s, 10% (2/11) of patients experimentally challenged with the Chesson strain of *P*. *vivax* and then treated with an 8 week regimen (~5.1 mg/kg total dose) relapsed, compared to 27% (16/60) of patients treated with 15 mg primaquine administered daily for 14 days (~3 mg/kg total dose) [12]. In another case series of patients treated with PQ8W, there were no recurrent infections over 24 months in 10 patients experimentally infected with a South Vietnam strain of *P*. *vivax*, although in the same study this rose to 1 in 3 of the patients experimentally infected with the Chesson strain [31]. In a clinical trial conducted in Iran, less than 2% (2/145) of patients treated with PQ8W had recurrent *P*. *vivax* over 12 months; the background risk of relapse in this area is unknown [16].

The current WHO guidelines recommend a daily dose of PQ in adults of 15 mg (0.25 mg/kg) or 30 mg (0.5 mg/kg) for the 14 day regimen and weekly 45 mg (0.75mg/kg) in the 8 week regimen. The weekly dose of PQ in our study was weight adjusted to provide a total mg/kg dose of approximately 7mg/kg (S4 Table) and this required individual doses ranging between 0.66 and 0.98 mg/kg. Higher individual doses are associated with greater gastrointestinal side effects, although these can mitigated by coadministration of food [32]. Despite the higher individual doses, PQ8W was generally well tolerated in the patients in our study. No patients vomited their medication within an hour of administration and tolerability reported from routine questionnaires were similar to that reported in the larger contemporaneous trial (Table 5).

The primary safety concern for primaquine relates to its ability to cause severe hemolysis in patients with G6PD deficiency [33,34]. Our observational study exposed patients with a range of G6PDd variants to weekly primaquine. The haematological profiles and recovery following malaria in patients with G6PDd treated with PQ8W were similar to that reported in G6PD normal patients treated with daily PQ, although G6PDd patients had a higher maximal fall in Hb (1.8g/dL compared to 0.8–1.0 g/dL, respectively), and a higher maximal fractional fall in Hb (13% compared to 6.4–7.4%, respectively); Table 4. [15]. Similar to the observations in the early experimental studies which led to the weekly regimen [12], we observed that most of the reduction in Hb occurred in the first week after starting treatment, likely exacerbated by parasite induced hemolysis [35].

Weekly spacing of PQ administration allows some haematological recovery between drug exposures. Two male patients with Mediterranean and Kaiping variants had a fall in Hb >5g/dL, but both started with high baseline Hb concentrations, neither had a fall in Hb below 10g/dL or reported concomitant symptoms, and both recovered quickly while completing their course of primaquine. By contrast, a 16-year old male with the Kaiping variant had a 33% fall in Hb to 9.0 g/dL, associated with general malaise, anorexia and reduced oral intake requiring intravenous fluid administration in hospital. A similar event occurred in a Cambodian trial, in which a male with the Viangchan variant was treated with PQ8W and had a fractional fall of 25% (from 10 to 7.5 g/dL) resulting in symptomatic anemia necessitating a blood transfusion [15]. Serious adverse events are an inherent risk of primaquine in areas where the more severe G6PD variants are prevalent and the decision to use this regimen must consider the benefits and how to minimise harm. It is important that patients are counselled when primaquine is prescribed by well-trained healthcare providers and are reminded of the importance of seeking urgent medical review if they experience symptoms suggestive of acute hemolysis. The risk of drug induced hemolysis in these patients should be balanced against the benefits of preventing recurrent *P*. *vivax* infections and cumulative risks of severe anemia associated with multiple relapses [36].

Our study has several limitations. We were unable to measure the enzyme activity by spectrophotometry at all sites and thus relied on screening with the FST. This qualitative assay only detects G6PD deficiency in patients with less than 30% enzyme activity and is vulnerable to subjective visual discrimination [21]. For example, in North Sumatra, where spectrophotometry was used, 8 of the 13 patients enrolled were found to have wild type genotypes with G6PD enzyme activity exceeding 60%, a likely reflection of inherent errors in interpreting the FST test. This over-representation of patients with relatively high G6PD activity may have biased our safety analysis. To address this, we undertook a subgroup analysis of patients in whom G6PDd could be confirmed by the presence of variants previously associated with significant reduction in enzyme activity. The overall safety profile in patients with genetically confirmed and unconfirmed G6PDd were similar (Table 4), however three of the 26 hemizygous males had significant falls in haemoglobin after the first dose of PQ; all tolerated their second dose of PQ and were able to complete treatment. The FST is not widely used in endemic settings, and when it is used is prone to errors in interpretation and thus potential to offer patients suboptimal treatment. Novel quantitative point of care tests are now available that have potential to improve safer and more effective radical cure options.

Another limitation of our analysis is the relatively low patient recruitment into the study. Despite being the largest study of primaquine in patients with G6PDd, the sample size is relatively small for an antimalarial clinical trial conducted over multiple sites. This limitation has confounded previous clinical trials seeking to enrol intermediate or severely G6PD deficient patients and likely reflects the protective effect of G6PDd against acute disease [28].

In conclusion, our study provides important new evidence on the tolerability, safety and efficacy of PQ8W in Asian and African patients with G6PDd. PQ8W was highly effective in preventing *P*. *vivax* recurrences. Although PQ8W is well tolerated in most patients, significant falls in haemoglobin can occur after the first dose highlighting the importance of clinical monitoring to identify vulnerable patients with early signs of hemolysis, before clinical deterioration. As routine point of care testing for G6PDd becomes more widely available, PQ8W offers relatively safe and effective radical cure for patients who are precluded from taking daily primaquine regimens or single dose tafenoquine.

## Supporting information

S1 Text Statistical analysis plan. (PDF)

S1 Table Literature review of clinical trials treating patients with weekly primaquine regimens. (DOCX)

S2 Table Information about study sites. (DOCX)

S3 Table List of Ethics Review Boards and Regulatory Agencies. (DOCX)

S4 Table Weekly primaquine dosing. (DOCX)

S5 Table Dosing table for dihydroartemisinin piperaquine used in Indonesia. (DOCX)

S6 Table Baseline Characteristics of all patients stratified by site. (DOCX)

S1 Data Study data. (XLSX)
