# Supplementary material for: Weekly primaquine for radical cure of patients with Plasmodium vivax malaria and glucose-6-phosphate dehydrogenase deficiency
Source: PLoS Negl Trop Dis. 2023 Sep 6;17(9):e0011522. doi: 10.1371/journal.pntd.0011522 (PMC10482257; doi:10.1371/journal.pntd.0011522)
Supplement: S1 Text — (PDF) [file pntd.0011522.s001.pdf]

Statistical Analysis Plan

G6PD deficient patients from Improving the radical cure of vivax malaria (IMPROV)

Version number: 1.0

Date: 8th September 2021

1 Introduction 2
2 Study design 2
3 Objectives 2
3.1 Primary Objectives 2
3.2 Secondary objectives 2
3.3 Sample size 2
4 Efficacy endpoints 3
4.1 Primary efficacy endpoint – radical cure 3
4.2 Secondary efficacy endpoints – radical cure 3
4.3 Secondary Efficacy endpoints – blood stage treatment 3
5 Safety Endpoints 3
5.1 Haematological endpoints 3
5.2 Adverse events 3
6 Definitions 4
6.1 Symptomatic patients 4
6.2 Study site 4
7 Analyses Populations 4
8 Analyses 4
8.1 Demographic and baseline characteristics 4
8.2 Adherence 5
8.3 Graphs .... **Error! Bookmark not defined.**
8.3.1 Kaplan Meier survival curve 5
8.3.2 Haemoglobin dynamics 5
8.3.2.1 Known G6PD variant 5
8.3.2.2 Unknown G6PD variants 6
9 Handling of missing data and adjudication of Endpoints 6
9.1 Handling of missing data on drug course 6
9.2 Handling of missing data for Adverse Events 6

9.3 Adjudication of day 42-365 efficacy outcome assessment.... 6
9.4 Adjudication of day 28 and 42 efficacy outcome assessment.... 7
9.5 Adjudication for endpoint on parasite prevalence on day 0-3.... 7
9.6 Adjudication for endpoint on fever clearance on day 0-3.... 7
9.7 Adjudication for haemoglobin outcome.... 7

1 Introduction

This statistical analysis plan (SAP) is a supplement to the main IMPROV SAP and is focused on the safety and efficacy of patients diagnosed with G6PD deficiency and treated with weekly primaquine.

2 Study design

This was an open label, single arm safety and efficacy study.

All subjects received the standard treatment for blood stage vivax, either chloroquine or an artemisinin based combination, dosed according to local guidelines. Primaquine was administered under supervision weekly (target dose 0.75 mg/kg/week) for 8 weeks, with a total target dose of 6 mg/kg.

Recurrences of any species within 28 days were considered treatment failures and treated with local second line alternatives (such as ACT or 7 days quinine), according to national guidelines. Any patients with *P. vivax* recurrence after 28 days, were retreated with the same regimen as at baseline.

2.1 Sample size

Sampling was opportunistic. All patients who were eligible for enrolment into the main study but excluded due to G6PD deficiency, were offered to be enrolled into the complementary study arm.

3 Objectives

3.1 Primary objectives

1. To determine the risk of recurrent *P. vivax* malaria following weekly primaquine in G6PDd patients.
2. To assess the safety of weekly primaquine radical cure.

3.2 Secondary objectives

1. To quantify the timing and frequency of *P. vivax* recurrence in G6PDd patients in different endemic settings.
2. To determine the early clinical and parasitological response following chloroquine or ACT and weekly primaquine.
3. To determine the haemoglobin profile and associated determinants following weekly primaquine.
4. Factors associated with haemoglobin recovery.

4 Efficacy endpoints

All efficacy endpoints refer to microscopically-proven recurrent parasitaemia, irrespective of symptoms i.e. symptomatic and asymptomatic recurrences will be combined.

4.1 Primary efficacy endpoint – radical cure

1. Incidence rate (per person year) of all recurrent *P. vivax* parasitaemia over 12 months of follow-up.

4.2 Secondary efficacy endpoints – radical cure

1. Incidence risk* of all recurrences of *P. vivax* over 12 months of follow up.
2. Incidence rate of any parasitaemia (all species) over 12 months of follow-up.
3. Incidence risk* of any parasitaemia (all species) over 12 months of follow-up.

* this is the conditional probability of recurrence at time t, given the individual has not had a recurrence prior to this time, calculated using the Kaplan-Meier method

4.3 Secondary efficacy endpoints – blood stage treatment

All secondary efficacy endpoints refer to the treatment outcome following the first episode of *P. vivax* infection (enrolment episode) or the first symptomatic episode of *P. falciparum*.

1. Incidence risk of *P. vivax* recurrence by D28.
2. Proportion of patients with *P. vivax* parasitaemia on days 1, 2 and 3.
3. Proportion of patients with fever on days 1, 2 and 3.

Handling of missing data and adjudication of endpoints is presented in section 9.

5 Safety Endpoints

5.1 Haematological endpoints

1. Incidence risk* of severe anaemia (Hb <7g/dL) and/or blood transfusion during first 63 days of follow up in patients presenting with Hb ≥7g/dL.
2. Incidence risk* of patients with an acute drop in Hb >5g/dL during the first 63 days of follow up.
3. Mean fall in Hb concentration from baseline on day 3 and day 7.
4. Nadir Hb concentration (defined as minimum Hb concentration measurement for a given individual over 63 days) and the median (IQR, range) time at which it occurs.
5. Maximal fractional change of Hb concentration, calculated from the difference in Hb at nadir from baseline.

* this is the conditional probability of severe anaemia or >5g drop at time t, given the individual has had at least one Hb measure per week to this time, calculated using the Kaplan-Meier method

5.2 Adverse events

All adverse events will be graded according to the toxicity table used in the study: National Institute of Allergy and Infectious Diseases Table for grading the severity of ADULT and PEDIATRIC adverse events.

http://www.niaid.nih.gov/LabsAndResources/resources/DMIDClinRsrch/pages/toxtables.aspx

The following AEs will be reported:

1. Within 1h of study drug administration, the proportions of participants who vomited
2. From day 1 – 14, the proportion of participants with:
    a. Nausea
    b. Vomiting
    c. Abdominal pain
3. Any AE leading to study drug withdrawal
4. All SAEs over 12 months irrespective of their relationship to study drugs

6 Definitions

6.1 Symptomatic patients

Symptomatic patients are defined as patients with *P. vivax* parasitaemia and either a documented fever (axillary temperature ≥37.5°C) or a history of fever within the preceding 48 hours.

6.2 Study site

A study site is defined as study location(s) under the responsibility of one local Principal investigator (PI) (e.g. one site could consist of two study hospitals in the same area under the responsibility of the same PI).

7 Analyses populations

All patients who received one dose of study drug will be included in the analysis of efficacy and safety.

A safety subgroup analysis will also be conducted in patients with confirmed G6PDd, determined by either spectrophotometry or genotyping.

8 Analyses

8.1 Demographic and baseline characteristics

A CONSORT flow diagram will be presented. The number of patients discontinuing from the study and for which reason/s will be tabulated. The baseline value is defined as the last available value before randomisation.

The following data will be presented in Table 1:

1. Gender: male; female
2. Age (years), median 25th-75th percentiles, and range
3. Age in classes: 12 months up to 5 years, 5 years up to 15 years, ≥15 years
4. Weight (kg), median, 25th-75th percentiles, and range
5. Weight in classes: <9kg; ≥9kg and <18kg; ≥18kg and <36 kg; ≥36 kg

Symptoms:

6. Fever
7. Headache
8. Dizziness
9. Myalgia (muscle/joint aches & pains)
10. Anorexia (poor appetite)
11. Nausea
12. Vomiting
13. Abdominal pain
14. Diarrhoea (watery or >3 stools per day)
15. Shortness of breath
16. Rash
17. Itching
18. Passing dark urine
19. Duration of illness

Signs:

20. Axillary temperature (°C), mean, SD
21. Percent febrile (temperature ≥37.5°C)

Laboratory results:

22. Asexual parasite count (parasites/µL), geometric mean, SD and 95% normal range
23. Presence of gametocytes
24. Gametocytes densities (parasites/µL), geometric mean, SD and 95% normal range
25. Haemoglobin concentration (g/dL), mean and SD
26. Anaemia (haemoglobin <10g/dL)

Treatment:

27. Primaquine dose received in mg/kg, median, 25th-75th percentiles, range

Genotype:

28. G6PD deficiency variant: AF1; AF2; Canton; Chatham; Gaohe; Kaipaing; Ludhiana; Med; Orissa; Viangchan; Viangchan/VanuaLava; +357A>G; Unknown
29. Hemizygous or heterozygous status

8.2 Adherence

The proportion of patients completing a full course of observed primaquine therapy will be calculated.

8.3 Plots

8.3.1 Kaplan Meier survival curve

This will show the cumulative risk of recurrence of *P. vivax* parasitaemia over 12 months.

8.3.2 Haemoglobin dynamics

8.3.2.1 Known G6PD variant

Separate plots for males and females showing individual Hb data over time – D0 to D63.

8.3.2.2 Unknown G6PD variants

Separate graphs for males and females showing individual Hb data over time – D0 to D63

An example is shown below of G6PDd and G6PD normal females (Taylor *et al*, submitted).

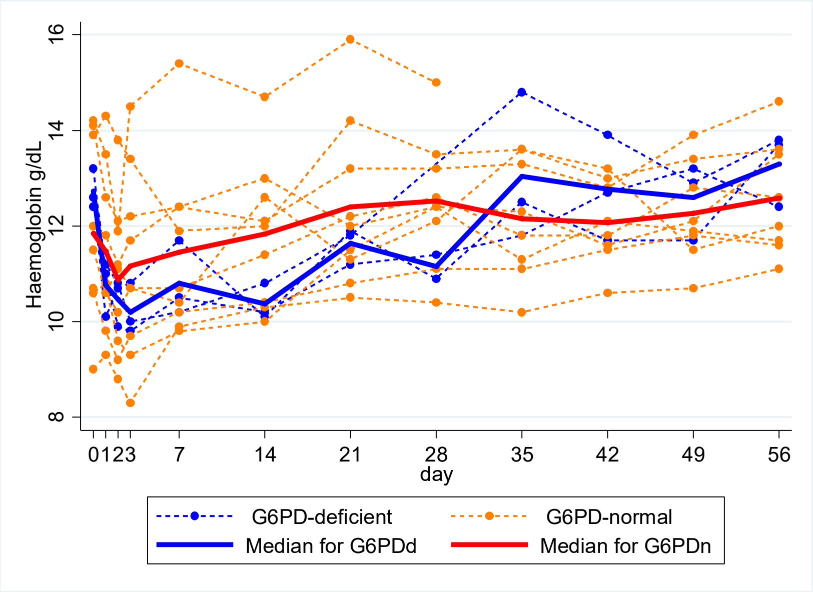

9 Handling of missing data and adjudication of endpoints

9.1 Handling of missing data on drug course

Patients can have an incomplete course of treatment or data on drug administration may be missing. No imputation of treatment course will be made for patients with missing data. Subgroup analyses will be performed for patients with full versus partial treatment courses (see section **Error! Reference source not found.**).

9.2 Handling of missing data for adverse Events

For patients with missing data on adverse events the most conservative approach will be used:

Deviation	Action
Start date of AE missing	Assume during study drug intake
End date of AE missing	No imputation
Date of start of study treatment administration missing	All AEs after randomization considered to have happened during study drug intake
Missing assessment of relationship to study treatment	Assume event to be possibly related
Missing severity assessment of AE	Assume highest severity

9.3 Adjudication of day 42-365 efficacy outcome assessment

For missed visits during the 1 year follow up, the following rules will apply:

Deviation	Action

More than 2 consecutive months without blood film examination	-In the survival analysis, lost to follow up (i.e. censored) on day of last visit before missing observation period.<br>-In the incidence rate analysis, the period of missing observation will be deducted from the total period of observation.

9.4 Adjudication of day 28 efficacy outcome assessment

The clinical definition of treatment failure enables determination of the efficacy endpoint for schizontocidal activity. However, when the number of scheduled visits is incomplete the following rules will be applied[1]:

Outcome assessment	Deviation	Action
**Day 28**	More than 18 days without blood smear results	Lost to follow up on the day of last observation
	No blood smear results between day 25 and day 31	Lost to follow up on day of last visit before day 25

9.5 Adjudication for endpoint on parasite prevalence on day 0-3

For missed blood films (microscopy) the following rules will be applied[1]:

Parasitaemia by microscopy				Action
**Day 0**	**Day 1**	**Day 2**	**Day 3**	
Yes	Yes	No	Missing	Assumed no parasitaemia on day 3
Yes	Yes	Missing	Yes	Assumed parasitaemia on day 2
Yes	No	Missing	Missing	Assumed no parasitaemia on day 2 and 3
Yes	Yes	Missing	No	Will not contribute to proportion of patients with parasitaemia on day 2
Yes	No	Missing	No	Assumed no parasitaemia on day 2
Yes	No	Missing	Yes	Will not contribute to proportion of day 2 with parasitaemia
Yes	Yes	Missing	Missing	Will not contribute to proportion of patients with parasitaemia on day 2 or 3

9.6 Adjudication for endpoint on fever clearance on day 0-3

For missed temperature records, the same rules as for missing blood slides will apply.

9.7 Adjudication for haemoglobin outcome

For missing haemoglobin measurements during follow up the following rules apply:

Outcome assessment	Deviation	Action

[1] WWARN. Clinical Module, Data Management and Statistical Analyses Plan. 2013; Available from: http://www.wwarn.org/sites/default/files/ClinicalDMSAP.pdf

Incidence risk of severe anaemia within first 63 days	More than one week (7 days) without a Hb measurement	Lost to follow up on day of last visit before missing observation period
Incidence risk of acute Hb drop within first 63 days	More than one week (7 days) without a Hb measurement	Lost to follow up on day of last visit before missing observation period
Hb concentration at day 3	Missing Hb measurement on day 3	Exclude from analysis
Hb concentration at day 7	Missing Hb measurement on day 7	Exclude from analysis
Hb nadir (during first 63 days)	2 or more consecutive scheduled visits within 63 days without Hb measurement	Excluded from analysis
