# Supplementary material for: Weekly primaquine for radical cure of patients with Plasmodium vivax malaria and glucose-6-phosphate dehydrogenase deficiency
Source: PLoS Negl Trop Dis. 2023 Sep 6;17(9):e0011522. doi: 10.1371/journal.pntd.0011522 (PMC10482257; doi:10.1371/journal.pntd.0011522)
Supplement: S1 Table — (DOCX) [file pntd.0011522.s002.docx]

## Supplementary Table 1 - Literature review of clinical trials treating patients with weekly primaquine regimens

All clinical trials including a treatment arm with weekly primaquine dosing for 8 weeks (total dose 6mg/kg). Derived from systematic review registered in PROSPERO [CRD42016053228]

|  |  | Duration of follow up  (Days) | Treatment arms | Number of patients treated with PQ8W | Phenotypically Confirmed G6PD deficiency | Primaquine Supervision | Comment |
| --- | --- | --- | --- | --- | --- | --- | --- |
| Azarian Moghadam et al Iranian Journal of Public Health - 2018 | Iran | 28 | CQ plus PQ8W.  No comparator Arm | 170 | Not Tested | Not stated | Antirelapse efficacy not assessed |
| Kheng et al  BMC Medicine - 2015 | Cambodia | 56 | DHP plus PQ8W  No comparator Arm | 75 | 18 | Supervised |  |
| Leslie et al  PLoS ONE -2008 | Pakistan | 365 | CQ plus PQ8W  Compared to PQ14 and Placebo arms | 74 | 1 | Supervised |  |
| Miahipour et al  Trans R Soc Trop Med Hyg - 2013 | Iran | 540 | CQ plus PQ8W  Compared to placebo arm | 160 | Not Tested | Not Stated | PQ started on day 3 |
| Nateghpour et al  Iranian Journal of Public Health - 2007 | Iran | 28 | CQ plus PQ8W  No comparator arm | 225 | Not Tested | Not Stated | PQ started on day 7, antirelapse efficacy not assessed |
| Shaikh Journal of Pharmaceutical Research International -2021 | Pakistan | 56 | AL plus PQ8W  No comparator arm | 40 | 40 | Not Stated |  |
| Than et al  Trans R Soc Trop Med Hyg - 1995 | Myanmar | 28 | CQ plus PQ8W  No comparator arm | 50 | Not Tested | Unsupervised | PQ started on Day 3 – antirelapse efficacy not assessed |
