# Supplementary material for: Weekly primaquine for radical cure of patients with Plasmodium vivax malaria and glucose-6-phosphate dehydrogenase deficiency
Source: PLoS Negl Trop Dis. 2023 Sep 6;17(9):e0011522. doi: 10.1371/journal.pntd.0011522 (PMC10482257; doi:10.1371/journal.pntd.0011522)
Supplement: S2 Table — (DOCX) [file pntd.0011522.s003.docx]

## Supplementary Table 2 - Information About Study Sites

|  | **Afghanistan** | | **Ethiopia** | | **Indonesia** | | **Vietnam** | |
| --- | --- | --- | --- | --- | --- | --- | --- | --- |
| **Site Name** | **Jalalabad** | **Laghman** | **Arba Minch** | **Metahara** | **Hanura** | **Tanjung Leidong** | **Dak O & Bu Gia Map** | **Krong Pa** |
| **Site Code** | AF001 | AF008 | ET001 | ET002 | ID004 | ID005 | VN001 | VN002 |
| **Name of study center** | Speen-Ghar Teaching Hospital | Laghman, Mihterlam hospital | Arba Minch Hospital | Metehara Sugar Factory Hospital | Hanura Primary Health Center | Tanjung Leidong | Dak-O & Bu Gia Map Commune Health Stations | Krong Pa Medical Center |
| **Study center GPS coordinates** | 34.4344/ 70.4612 | 34.6891/ 70.1467 | 6.025204/ 37.556696 | 8.8875/ 39.9169444 | -5.320619/ 105.622958 | 2.698056/ 99.901667 | 12.047594/ 107.093178 | 13.221819/ 108.682475 |
| **Altitude above sea level** | 575 m | 779m | 1285m | 946 m | 15 – 65 m | 5m | 338-408 m | 136 m |
| **Treatment policy (incl. G6PD testing)** | **Pf:** AL+PQ (single dose 0.25 mg/kg)  **Pv:** CQ+PQ (0.25 mg/kg for 14 days)  **G6Pd testing** recommended. If not available, weekly PQ course recommended | | **Pf:** AL+PQ (single dose 0.25 mg/kg)  **Pv:** CQ+PQ (0.25 mg/kg for 14 days)  **G6PD testing** not explicitly recommended | | **Pf:** DHP+PQ (single dose 0.25 mg/kg)  **Pv:** DHP+PQ (0.25 mg/kg for 14 days).  **G6PD testing** not explicitly recommended | | **Pf**: DHP+PQ (single dose 0.25 mg/kg)  **Pv**: CQ+PQ  (0.25 mg/kg for 14 days)  **G6PD testing** not explicitly recommended | |
| **G6PD prevalence and variants** | Mediterranean variant 7% in many ethnic groups^[[1]](#footnote-1)^ | Mediterranean variant 7% in many ethnic groups^1^ | Unknown, likely A- and Mediterranean | Unknown, likely A- and Mediterranean | Unknown | 2.7%, Mahidol variant^[[2]](#footnote-2)^ | Viangchan, Canton, Kaiping, Union  (Kinh = 2%^[[3]](#footnote-3)^, other ethnicities 10%^[[4]](#footnote-4)^) | |
| **Approx. catchment population of study center** | 356,274 | 100,000 | 164,529 | 22,0259 | 35,692 | 21,191 | 22,000 | 70,000 |
| **Seasonality of malaria** | May to Nov (peak in summer /July), Pf is confined to Oct-Nov) | May to Nov (peak in summer /July), Pf is confined to Oct-Nov) | Year round with peak at Dec- Feb | Peak malaria Sept-October, smaller season April | Peak season November and march | Year round, peak between July-December | Peak malaria season  October to April | Peak malaria season  October to January |
| **P.v./ P.f. ratio** | 95:5 | 90:10 | 30:70 | 40:60 | 40:60 | 95:5 | 40:60 | 40:60 |
| **Relapse periodicity** |  |  | 3-5 months | 3-5 months^[[5]](#footnote-5)^ | 3 months | 3 months | 45 days | 3 months |
| **Vectors** | *An.stephensi*  *An.culicifacies*  *An. fluviatilus*  *An.annularis*  *An.pulcherrimus*  *An.superpictus*  *An.hycranus* | An.stephensi  An.subpictus  An.culicifacies | *An.arabiensis* | *An.arabiensis* | *An.sundaicus* | Not known, probably  *An. sundaicus* | *An.dirus*  *An.minimus* | *An.dirus*  *An.minimus* |
| **API** | 3.5 per 1,000 per year^[[6]](#footnote-6)^ | 1.62 per 1,000 per year | unknown | unknown | 43.9 per 1,000 per year | 49 per 1,000 per year^[[7]](#footnote-7)^ | 50 per 1,000 year^[[8]](#footnote-8)^ | 8.5 per 1,000 year^6^ |

1. CQ = Chloroquine; PQ = primaquine; DHP = Dihydroartemisinin-piperaquine; AL = artemether-lumefantrine

   Jamornthanyawat N*et al* A Population Survey of the Glucose-6-Phosphate Dehydrogenase (G6PD) 563C>T (Mediterranean) Mutation in Afghanistan. *PLoS One* 2014; 9(2):e88605. [↑](#footnote-ref-1)
2. Pasaribu *et al.* A randomised comparison of dihydroartemisinin-piperaquine and artesunate-amodiaquine combined with primaquine for radical treatment of vivax malaria in Sumatera, Indonesia. *J Infect Dis* 2013 ;208(11):1906-13. [↑](#footnote-ref-2)
3. Matsuoka et al. Seven different glucose-6-phosphate dehydrogenase variants including a new variant distributed in Lam Dong Province in southern Vietnam. Acta Med Okayama. 2007;61(4):213-9 [↑](#footnote-ref-3)
4. Bancone et al. Molecular characterization and mapping of glucose-6-phosphate dehydrogenase (G6PD) mutations in the Greater Mekong Subregion. Malar J. 2019;18(1):20 [↑](#footnote-ref-4)
5. Abreha *et al*. Comparison of artemether-lumefantrine and chloroquine with and without primaquine for the treatment of Plasmodium vivax infection in Ethiopia: A randomised controlled trial. *PLoS Med* 2017 ;14(5):e1002299. doi: 10.1371/journal.pmed.1002299 [↑](#footnote-ref-5)
6. Based on the national strategic plan for malaria control to elimination in Afghanistan 2018-2022 by the Ministry of Public Health, Afghanistan [↑](#footnote-ref-6)
7. Based on data from the Ministry of Health on annual parasite incidence (API) in 2016 [↑](#footnote-ref-7)
8. Based on reports of the National Malaria Control Program (NMCP of Vietnam) in 2017 [↑](#footnote-ref-8)
