# Supplementary material for: Weekly primaquine for radical cure of patients with Plasmodium vivax malaria and glucose-6-phosphate dehydrogenase deficiency
Source: PLoS Negl Trop Dis. 2023 Sep 6;17(9):e0011522. doi: 10.1371/journal.pntd.0011522 (PMC10482257; doi:10.1371/journal.pntd.0011522)
Supplement: S3 Table — (DOCX) [file pntd.0011522.s004.docx]

## Supplementary Table 3 - List of Ethics Review Boards and Regulatory Agencies

| **Australia** | **The Human Research Ethics Committee of the Northern Territory Department of Health (HREC)**  John Mathews Building (Bldg 58)  Royal Darwin Hospital Campus, Rock  PO Box: 41096, Casuarina NT 0811, Australia  Website: www.menzies.edu.au |
| --- | --- |
| **United Kingdom** | **The Oxford Tropical Research Ethics Committee (OxTREC )**  University of Oxford  Research Services, University Offices  Willington Square, Oxford OX1 2JD  Tel: +44 (0) 1865 (2) 82106  E-mail: [oxtrec@admin.ox.ac.uk](mailto:oxtrec@admin.ox.ac.uk)  Website: [www.admin.ox.ac.uk/rso/](http://www.admin.ox.ac.uk/rso/) |
| **Afghanistan** | **Islamic Republic of Afghanistan, Ministry of Public Health, Institutional Review Board**  3^rd^ floor of Main Building of Ministry of Public Health  Masoud Circle, Wazir Akbar Khan, Kabul-Afghanistan  Tel: +93(0) 20 29 22 435  email: [anphi@moph.gov.af](mailto:anphi@moph.gov.af) |
| **Ethiopia** | **The National Research Ethics Review Committee (NRERC)**  Addis Ababa, Ethiopia  PO Box: 2490  Tel: +251 114-674-353  E-mail: [most@ethionet.et](mailto:most@ethionet.et)  Fax: +251 114-660-241  Website: [www.most.gov.et](http://www.most.gov.et) |
|  | **Scientific & Ethical Review Committee (SERC)**  Ethiopian Public Health Institute  Addis Ababa, Ethiopia  PO Box: 1242/5654  E-mail: [ephi@ethionet.et](mailto:ephi@ethionet.et)  Tel: +251 11 2133499, +251 11 2751522  Fax: +251 11 2758634  Website: [www.ephi.gov.et](http://www.ephi.gov.et) |
|  | **The Food Medicine and Health Care Administration and Control Authority (FMHACA)**  Addis Ababa, Ethiopia  Tel: 251-11-552 41 22/552 41 23  E-mail: [regulatory@fmaca.gov.et](mailto:regulatory@fmaca.gov.et)  Fax: 251-11-552 13 92  PO Box: 5681 |
| **Indonesia** | **The Health Research Ethics Committee of the Faculty of Medicine University of Indonesia**  Cipto Mangunkusumo Hospital  Jalan Salemba Raya No. 6, Jakarta Pusat 10430  Tel: 021-3157008  E-mail: [ec_fkui@yahoo.com](mailto:ec_fkui@yahoo.com) |
|  | **Indonesian Food and Drug Agency (BPOM)**  Jl, Percetakan Negara No. 23 Jakarta Pusat 10560 Indonesia  E-mail: [infopom@indo.net.id](mailto:infopom@indo.net.id);  Tel: (021) 4244691, 4209221, 4263333, 4244755, 4241781, 4244819  Fax: (02) 4245139  Website: [www.pom.go.id](http://www.pom.go.id) |
| **Vietnam** | **The Ministry of Health Evaluation Committee on Ethics in Biomedical Research**  138A Giang Vo Street, Ba Dinh District, Ha Noi, Viet Nam  Email: iecmoh@gmail.com  Tel: +84 4 6273 2156 |
