# Supplementary material for: Weekly primaquine for radical cure of patients with Plasmodium vivax malaria and glucose-6-phosphate dehydrogenase deficiency
Source: PLoS Negl Trop Dis. 2023 Sep 6;17(9):e0011522. doi: 10.1371/journal.pntd.0011522 (PMC10482257; doi:10.1371/journal.pntd.0011522)
Supplement: S4 Table — (DOCX) [file pntd.0011522.s005.docx]

## Supplementary Table 4 - Weekly Primaquine Dosing

In children under 23 kgs, 7.5 mg and 15 mg tablets were dissolved in 5 mL of syrup to produce 4.5mg per 1ml

| **Wt in kg** | **ml/day** | **Dose per week**  **mg** | **Dose per week**  **mg/kg** | | **Total Dose**  **mg/kg** | |
| --- | --- | --- | --- | --- | --- | --- |
| Suspension | | |  | |  | |
| 5 | 0.8 | 3.60 | 0.72 | | 5.76 | |
| 6 | 1 | 4.50 | 0.75 | | 6.00 | |
| 7 | 1 | 4.50 | 0.64 | | 5.14 | |
| 8 | 1.25 | 5.63 | 0.70 | | 5.63 | |
| 9 | 1.5 | 6.75 | 0.75 | | 6.00 | |
| 10 | 1.5 | 6.75 | 0.68 | | 5.40 | |
| 11 | 1.75 | 7.88 | 0.72 | | 5.73 | |
| 12 | 2 | 9.00 | 0.75 | | 6.00 | |
| 13 | 2 | 9.00 | 0.69 | | 5.54 | |
| 14 | 2.5 | 11.25 | 0.80 | | 6.43 | |
| 15 | 2.5 | 11.25 | 0.75 | | 6.00 | |
| 16 | 2.5 | 11.25 | 0.70 | | 5.63 | |
| 17 | 3 | 13.50 | 0.79 | | 6.35 | |
| 18 | 3 | 13.50 | 0.75 | | 6.00 | |
| 19 | 3 | 13.50 | 0.71 | | 5.68 | |
| 20 | 3 | 13.50 | 0.68 | | 5.40 | |
| 21 | 3.5 | 15.75 | 0.75 | | 6.00 | |
| 22 | 3.5 | 15.75 | 0.72 | | 5.73 | |
| **Tablets** | | | **Min** | **Max** | **Min** | **Max** |
| 23-34 | - | 22.5 | 0.66 | 0.98 | 5.28 | 7.84 |
| 35-45 | - | 30 | 0.67 | 0.86 | 5.36 | 6.88 |
| 46 - 84 | - | 45 | 0.54 | 0.98 | 4.32 | 7.84 |
