# Supplementary material for: Weekly primaquine for radical cure of patients with Plasmodium vivax malaria and glucose-6-phosphate dehydrogenase deficiency
Source: PLoS Negl Trop Dis. 2023 Sep 6;17(9):e0011522. doi: 10.1371/journal.pntd.0011522 (PMC10482257; doi:10.1371/journal.pntd.0011522)
Supplement: S5 Table — (DOCX) [file pntd.0011522.s006.docx]

## Supplementary Table 5 - Dosing table for dihydroartemisinin piperaquine used in Indonesia.

Each tablet of dihydroartemisinin piperaquine contains 40 mg of dihydroartemisinin and 320 mg of piperaquine.

| **Weight**  **(kg)** | **Number of tablets** | **DHA**  **mg/kg/day** | | **Piperaquine**  **mg/kg/day** | |
| --- | --- | --- | --- | --- | --- |
|  |  | **Min** | **Max** | **Min** | **Max** |
| 6 to10 | 0.25 | 1.00 | 1.67 | 8.00 | 13.33 |
| 11 to 17 | 0.5 | 1.18 | 1.82 | 9.41 | 14.55 |
| 18 to 30 | 1 | 1.33 | 2.22 | 10.67 | 17.78 |
| 31 to 40 | 2 | 2.00 | 2.58 | 16.00 | 20.65 |
| 41 to 59 | 3 | 2.03 | 2.93 | 16.27 | 23.41 |
| 60 to 84 | 4 | 1.90 | 2.67 | 15.24 | 21.33 |
