# Supplementary material for: Weekly primaquine for radical cure of patients with Plasmodium vivax malaria and glucose-6-phosphate dehydrogenase deficiency
Source: PLoS Negl Trop Dis. 2023 Sep 6;17(9):e0011522. doi: 10.1371/journal.pntd.0011522 (PMC10482257; doi:10.1371/journal.pntd.0011522)
Supplement: S6 Table — (DOCX) [file pntd.0011522.s007.docx]

## Supplementary Table 6 - Baseline Characteristics of all patients stratified by site

|  | **Afghanistan** | **Ethiopia** | **Indonesia** | **Vietnam** | **Total** |
| --- | --- | --- | --- | --- | --- |
|  | **N=6** | **N=5** | **N=19** | **N=20** | **N=50** |
| Age in years, median  (IQR)  [range] | 11.0  (4.0-19.0)  [0.0 - 60.0] | 21.0  (19.0-27.0) [16.0 - 35.0] | 23.0  (9.0-41.0)  [4.0 - 52.0] | 26.0  (18.0-38.0)  [3.0 - 56.0] | 23.0  (14.0-38.0)  [0.0 - 60.0] |
| **Age category*** months (m) or years(y) | | | | | |
| 6-11 m | 1 (16.7%) | 0 (0.0%) | 0 (0.0%) | 0 (0.0%) | 1 (2.0%) |
| ≥1 & <5 y | 1 (16.7%) | 0 (0.0%) | 1 (5.3%) | 1 (5.0%) | 3 (6.0%) |
| ≥5 & <15 y | 2 (33.3%) | 0 (0.0%) | 6 (31.6%) | 2 (10.0%) | 10 (20.0%) |
| ≥15 y | 2 (33.3%) | 5 (100.0%) | 12 (63.2%) | 17 (85.0%) | 36 (72.0%) |
| **Sex** | | | | | |
| Male | 6 (100.0%) | 5 (100.0%) | 14 (73.7%) | 17 (85.0%) | 42 (84.0%) |
| Female | 0 (0.0%) | 0 (0.0%) | 5 (26.3%) | 3 (15.0%) | 8 (16.0%) |
| **Weight** in kg median (IQR) | 32.0  (14.0-60.0) | 56.0  (49.5-58.0) | 48.8  (24.9-70.9) | 52.0  (45.5-59.5) | 51.0  (36.0-60.3) |
| **Weight category*** | | | | | |
| 10-22 kg | 3 (50.0%) | 0 (0.0%) | 3 (15.8%) | 1 (5.0%) | 7 (14.0%) |
| 23-34 kg | 0 (0.0%) | 0 (0.0%) | 3 (15.8%) | 1 (5.0%) | 4 (8.0%) |
| 34-45 kg | 0 (0.0%) | 1 (20.0%) | 2 (10.5%) | 3 (15.0%) | 6 (12.0%) |
| 46+ kg | 3 (50.0%) | 4 (80.0%) | 11 (57.9%) | 15 (75.0%) | 33 (66.0%) |
| ***P. vivax* parasites/uL** geometric mean (95% normal range) | 1118  (167 - 3333) | 6156 (1148 - 122500) | 2344  (37 - 14522) | 4073  (194 - 41079) | 2947  (70 - 25000) |
| **Gametocytaemia*** | 6 (100.0%) | 5 (100.0%) | 12 (63.2%) | 14 (70.0%) | 37 (74.0%) |
| **Gametocytes/uL** geometric mean (95% normal range) | 306.3  (55.6 – 1204) | 317.4  (55.6 – 2500) | 104.7  (7.4 – 600) | 92.7  (15.0 – 3833) | 138.2  (15.0 – 3593) |
| **Temperature** (˚C) Mean (95% CI)† | 36.9 (36.2 - 37.5) | 36.7 (35.8 - 37.6) | 37.3 (36.8 - 37.9) | 38.6 (38.2 - 39.1) | 37.7 (37.4 – 38.1) |
| **Fever** (Axillary >37.5C or Oral >38C) N (%) | 1 (16.7%) | 0 (0.0%) | 7 (36.8%) | 17 (85.0%) | 25 (50.0%) |
| **Hemoglobin** (g/dL) Mean (95% CI)† | 13.0 (9.5 - 16.5) | 13.8 (12.6 - 15.1) | 13.5 (12.4 - 14.6) | 13.5 (12.9 - 14.1) | 13.5 (12.9 – 14.0) |
| **Hb** <10 g/dL* | 1 (16.7%) | 0 (0.0%) | 2 (10.5%) | 0 (0.0%) | 3 (6.0%) |

* categorial data are N (%). † CI: Confidence Interval. Baseline data for the treatment arms in the randomised controlled trial are presented in Supplementary Table 5
